# Prenatal treatment with preimplantation factor improves early postnatal neurogenesis and cognitive impairments in a mouse model of Down syndrome

**DOI:** 10.1007/s00018-024-05245-9

**Published:** 2024-05-13

**Authors:** Manon Moreau, Rodolphe Dard, Amélia Madani, Janany Kandiah, Nadim Kassis, Jessica Ziga, Héloïse Castiglione, Solenn Day, Thomas Bourgeois, Boris Matrot, François Vialard, Nathalie Janel

**Affiliations:** 1 Université Paris Cité, BFA, UMR 8251, CNRS, Paris, F-75013 France; 2 Université Paris-Saclay, UVSQ, INRAE, ENVA, BREED, Jouy-en-Josas, 78350 France; 3 Département de Génétique, CHI de Poissy St Germain en Laye, Poissy, 78300 France; 4 NeuroDiderot, INSERM, Université Paris Cité, Paris, F-75019 France; 5 Laboratoire BFA, Université Paris Cité, 3 rue Marie-Andrée Lagroua Weill Hallé, Case, Paris cedex 13, 7104, F-75205 France

**Keywords:** Cyclin D1, c-myc, S100 beta, Dp(16)1Yey mice, Social behavior, Vocalizations

## Abstract

Down syndrome (DS) is a genetic disease characterized by a supernumerary chromosome 21. Intellectual deficiency (ID) is one of the most prominent features of DS. Central nervous system defects lead to learning disabilities, motor and language delays, and memory impairments. At present, a prenatal treatment for the ID in DS is lacking. Subcutaneous administration of synthetic preimplantation factor (sPIF, a peptide with a range of biological functions) in a model of severe brain damage has shown neuroprotective and anti-inflammatory properties by directly targeting neurons and microglia. Here, we evaluated the effect of PIF administration during gestation and until weaning on Dp(16)1Yey mice (a mouse model of DS). Possible effects at the juvenile stage were assessed using behavioral tests and molecular and histological analyses of the brain. To test the influence of perinatal sPIF treatment at the adult stage, hippocampus-dependent memory was evaluated on postnatal day 90. Dp(16)1Yey pups showed significant behavioral impairment, with impaired neurogenesis, microglial cell activation and a low microglial cell count, and the deregulated expression of genes linked to neuroinflammation and cell cycle regulation. Treatment with sPIF restored early postnatal hippocampal neurogenesis, with beneficial effects on astrocytes, microglia, inflammation, and cell cycle markers. Moreover, treatment with sPIF restored the level of DYRK1A, a protein that is involved in cognitive impairments in DS. In line with the beneficial effects on neurogenesis, perinatal treatment with sPIF was associated with an improvement in working memory in adult Dp(16)1Yey mice. Perinatal treatment with sPIF might be an option for mitigating cognitive impairments in people with DS.

**Supplementary Information:**

The online version contains supplementary material available at 10.1007/s00018-024-05245-9.

## Introduction

Down syndrome (DS, defined by the presence of an extra copy of chromosome 21) is the most common genetic syndrome in the general population. The clinical manifestations of DS vary from one individual to another and as a function of the environment, medical follow-up, and learning. The disability is manifested by growth disorders, insufficient muscle tone, delayed psychomotor development, cardiac and/or digestive malformations, morphological signs, and intellectual disability (ID). Furthermore, people with DS have a higher risk of developing neurodegenerative diseases, such as Alzheimer’s disease.

ID is the most serious problem for people with DS and has a major impact on families and society. For many years, it has been known that people with DS have fewer brain neurons; this relative decrease might be related to low neurogenesis, an abnormal neuron/astrocyte ratio, and excessive apoptosis in the brain [1]. Mouse models of DS show prenatal brain abnormalities, with a decrease in proliferation and neurogenesis [2, 3]. These abnormalities were also observed in the brain of human fetuses with DS [1, 4, 5]. The processes that control the number of brain neurons after successive waves of migration and differentiation occur before birth and after birth (during the first postnatal year, in humans). In both humans and mice, changes in inhibitory and excitatory neurons, synaptic dysfunction, and motor and cognitive impairments occur in the immediate postnatal period and during adulthood [6, 7].

Worldwide, intense research efforts are being focused on interventions that could mitigate or even prevent ID in DS. Although many different therapeutic approaches have been suggested, few are in clinical development [8–11]. All these approaches target a single pathophysiologic pathway in children and/or adults with DS. Since the neuronal alterations manifest themselves before birth, one can hypothesize that effective treatment during the prenatal period of brain development can fully rescue DS-related brain abnormalities. The administration of a molecule with positive effects on embryonic development would therefore be appropriate.

PreImplantation Factor (PIF, a peptide of embryonic origin) protects embryos against oxidative stress and protein misfolding [12] and reduces systemic levels of inflammatory cytokines in experimental model of prenatal inflammation [13]. Apart from having an essential role during gestation [14, 15], PIF also exerts neuroprotective properties by directly targeting microglia and neurons and promoting endogenous neural stem cell proliferation and the differentiation of remyelinated cells [16, 17]. These actions have been evidenced from newborns to adults [18, 19]. In human endometrial stromal cells, PIF promotes neural differentiation, axon guidance, and neurogenesis pathways involved in childhood-onset neurodevelopmental diseases. The primary objective of the present study was therefore to determine the putative neuroprotective effects on neurogenesis and neuroinflammation of prenatal and perinatal treatment with PIF in juvenile Dp(16)1Yey mice, a mouse model of DS which contains a duplication carrying 113 genes orthologous to genes on chromosome 21. The juvenile Dp(16)1Yey mice were obtained by crossbreeding C57BL6/J females with Dp(16)1Yey males to avoid the potential confound of a trisomic maternal uterine environment. The secondary objective was to assess adult mice for the persistence of any changes induced by this prenatal and perinatal treatment.

## Materials and methods

### Animals

Dp(16)1Yey mice [20] carry a duplication of the Lipi-Zfp295 region of the murine chromosome 16 syntenic to Hsa21. C57BL6/J females were crossbreed with Dp(16)1Yey males and thus produced litters containing both trisomic (Dp(16)1Yey) and wild-type (WT) offspring. Each pup’s bodyweight was measured from postnatal day 5 (P5) until sacrifice. The pups were genotyped as described elsewhere [20]. The mice were housed in controlled temperature (21 ± 1 °C) and humidity (55 ± 10%) conditions, with a 12 h/12 h light/dark cycle. Food and water were available *ad libitum*.

### Treatment with synthetic PIF

Gestating females were treated daily with a subcutaneous injection of 1.5 mg/kg synthetic PIF (sPIF, MVRIKPGSANKPSDD, BIOTEM) or vehicle (saline) from embryonic day 14 until weaning of the pups.

### Ultrasonic vocalizations

Ultrasonic vocalizations (USVs) were recorded on P7 for 3 min. The pup was isolated from the litter and placed inside a container (H 4.5 cm × L 10 cm × W 10 cm), which in turn was placed inside a soundproof chamber (H 23 x L 37 x W 24 cm) held at a temperature of 22 °C. The ultrasound microphone (Noldus, frequency range: 30–90 kHz) was placed at 10 cm above the pup. USVs were recorded with UltraVox XT3.1 software and analyzed using the Icy community platform provided by the Institut Pasteur and France Bioimaging, as described elsewhere [21]. The investigator who analyzed the recordings was blinded to the experimental group.

### Homing test

The pups’ nest odor recognition was evaluated on P8. The measurement platform (L 20 cm × W 13 cm) was composed of three zones (each L 7 cm × W 13 cm): the nest zone (NZ, containing litter from the pup’s original cage), the neutral zone (empty zone), and the clean zone (CZ, clean litter). The isolated P8 pup was placed in the neutral area. The camera (placed 30 cm above) recorded the pup’s displacements three times for 1 min. Homing performance was measured using Ethovision XT11 software (Noldus) and considering the distance moved (in cm), the time spent in the NZ and in the CZ (in sec), and the time needed to reach the NZ (in sec). The investigators analyzing the recordings were blinded to the experimental group.

### The Y-maze

Working memory was determined by measuring spontaneous alternation (SPA) in a Y-shaped maze. The Y-maze’s arms (labelled A, B and C) were 35 cm long, 5 cm wide and 15 cm tall. To avoid olfactory bias, the maze was cleaned between each experiment mouse. A camera was placed above the maze. On P90, mice were weighed and acclimated to the room 2 h before the test. The mouse was placed at the far end of the A arm, and the experimenter leave the testing room. The mouse was recorded for 10 min. The investigator was blinded to the experimental group. Arm entries (corresponding to all four paws in the arm) were noted, and SPA was scored when the animal entered the three different arms consecutively. Mice were killed by cervical dislocation at P100. Brains were harvested by dissection and weighed.

The proportion of SPAs (in %) was calculated as [(number of SPAs)/(total number of arm entries – 2)] x 100. Furthermore, the total number of arm entries was considered as an index of locomotor activity [22]. Time spent in the center of the maze was also measured. The investigator was blinded to the experimental group. 

### Protein extraction and immunoblotting

Mice were killed by decapitation. Brains were harvested by dissection, weighed, frozen in liquid nitrogen, and stored at -80 °C. Total protein extracts from whole brain were prepared in PBS containing protease inhibitors (1 mM Pefabloc SC, 5 µg/ml E64, and 2.5 µg/ml leupeptin). Immunoblotting was performed in accordance with standard Western blot or slot-blot protocols, depending on the antibody’s specificity. After transfer, membranes were saturated by incubation in 10% w/v non-fat milk powder or 5% w/v bovine serum albumin in Tris-saline buffer (1.5 mM Tris base, pH 8; 5 mM NaCl; 0.1% Tween-20), and incubated overnight at 4 °C with an antibody against DYRK1A (1/2000, H00001859-M01, Abnova Corporation), cyclin D1 (1:5000, ab134175, Abcam), p-cyclin D1 (T286) (1:1000, ab62151, Abcam), GSK3β (1:10000, ab93926, Abcam), p-GSK3β/α (S9/S21) (1:3000, ab226877, Abcam), p-GSK3β/α (Y216/Y279) (1:7000, ab75745, Abcam), mTOR (1:5000, ab137341, Abcam), p-mTOR (S2481) (1:5000, ab137133, Abcam), c-myc (1:2000, sc-40, Santa Cruz), p-c-myc (S62) (1:10000), ab185656, Abcam) or p-myc (T58) (1:9000, ab185655, Abcam). The immunoreactions were visualized with an Amersham ECL system (Amersham). Digitized images of immunoblots were obtained using a LAS-3000 imaging system (Fuji Photo Film Co. Ltd.), and densitometry data were collected with an image analyzer (UnScan It software, Silk Scientific Inc.). Total protein after Ponceau-S staining was used as an internal control. The levels of brain-derived neurotrophic factor (BDNF) in brain lysates were measured with a mouse BDNF Picokine™ ELISA kit (Boster).

### mRNA extraction, reverse transcription, and quantitative real time-PCR

Mice were killed by decapitation. Brains were harvested by dissection, weighed, frozen in liquid nitrogen, and stored at -80 °C. Total RNA was isolated from brains using a RNeasy Lipid Kit (Qiagen, Hilden, Germany). The RNA concentration was determined by measuring the optical density (OD) at 260 nm. The quality of the RNA was checked via the OD_260 nm_/OD_280 nm_ ratio. To remove residual DNA contamination, the RNA samples were treated with RNAse-free DNAse (Qiagen) and purified on a RNeasy mini column (Qiagen). For each sample, 4 µg of total RNA from each sample was reverse-transcribed using 200 U of M-MLV reverse transcriptase (Invitrogen, Life Technologies, Waltham, MA, USA) and random hexamer primers. Real-time quantitative PCR amplification reactions were carried out in a LightCycler 480 detection system (Roche, Basel, Switzerland), using the LightCycler FastStart DNA Master plus SYBR Green I Kit (Roche). Primer sequences are given in Table S1. For each reaction, 40 ng of reverse-transcribed RNA were used as a template. All reactions were carried out in duplicate, with a no-template control. The PCR conditions were 95 °C for 5 min, followed by 45 cycles of 95 °C for 10 s, 60 °C for 10 s and 72 °C for 10 s. The mRNA transcript level was normalized against the mean value for the genes *H1a* and *Tbp.* The target gene level was quantified using a method described elsewhere [23].

### Immunofluorescence and cell quantification

On P10, all pups received an injection of 50 mg/kg BrdU. Twenty-four hours after injection, the mice were killed by decapitation. The brain was removed from the skull, post-fixed in 4% paraformaldehyde (PFA) at 4 °C for 24 h, dehydrated in 15% sucrose and then 30% sucrose at 4 °C for 24 h, embedded in optimal cutting temperature compound medium, and stored at -80 °C. Serial coronal sections (thickness: 20 μm) were obtained using a freezing microtome (Leica CM3050 S) and stored at -80 °C before immunostaining. The cryosections were washed in PBS, incubated with 2 N HCl for 1 h and then with 0.1 M sodium borate for 30 min at room temperature (RT), washed three times in PBS, and blocked in PBS containing 0.3% Triton and 5% bovine serum albumin. After three washes in PBS, sections were double-labelled with the primary antibodies rat anti-BrdU (1:800, ab6326, Abcam) and rabbit anti-NeuN (1:1000, ab177487, Abcam) or rabbit anti-Iba1 (1:400, ab178846, Abcam) or rabbit anti-S100β (1:400, ab52642, Abcam). The sections were washed three times before incubation with the secondary antibodies Alexa Fluor 555-conjugated donkey anti-rabbit (1:800, A31572, Invitrogen) and Alexa Fluor 488-conjugated goat anti-rat (1:400, ab150157, Abcam) at RT for 3 h. Next, the sections were mounted in aqueous mounting medium with DAPI (ab104139, Abcam). Images of the somatosensory cortex and the dentate gyrus of bregma − 1.9 to − 2.3 were acquired with an IX83 Olympus fluorescence microscope equipped with a 20x objective, and CellSens software. A XY motorized stage allowed the acquisition of tiled images of the whole brain section. The regions of interest and the surface area were determined by using ImageJ v1.53t (Rasband, W.S., National Institutes of Health, Bethesda, Maryland). All the variables were quantified using Imaris software (version 9.9, Bitplane, Belfast, UK). Cells were counted with the Imaris Spot tool. The proliferation of neurons, astrocytes or microglia was determined by colocalization of NeuN, S100β or Iba1 staining with BrdU staining, using the Imaris Colocalization tool. The results were normalized against the surface area of the regions of interest. The length of the microglial (Iba1+) cell branches was analyzed using the Imaris Filament tool. The investigators were blinded to the experimental group.

### Statistical analysis

Statistical analyses were performed with GraphPad Prism software (version 8). All the data were analyzed in a three-way analysis of variance (ANOVA) to check interactions between the genotype (WT, Dp(16)1Yey), treatment (vehicle, sPIF) and sex (male, female). When no sex effect was found, data from males and females were pooled, and the genotype and treatment were analyzed in a two-way ANOVA. If an interaction was found, Fisher’s test was used when there were fewer than 10 samples per group, and the Bonferroni/Dunn test was used when there were 10 or more samples per group. In the absence of an interaction between the genotype and treatment factor, pairwise comparisons of groups used an unpaired t-test or the Mann-Whitney rank sum test, as appropriate. The threshold for statistical significance was set to *p* < 0.05. Given the small sample size, a p value of 0.06–0.10 was considered to indicate a strong statistical trend. Since the data were not normally distributed, correlations were analyzed by calculating Spearman’s rank correlation coefficient.

## Results

### Treatment with sPIF does not worsen the survival of Dp(16)1Yey pups

**Fig. 1:**
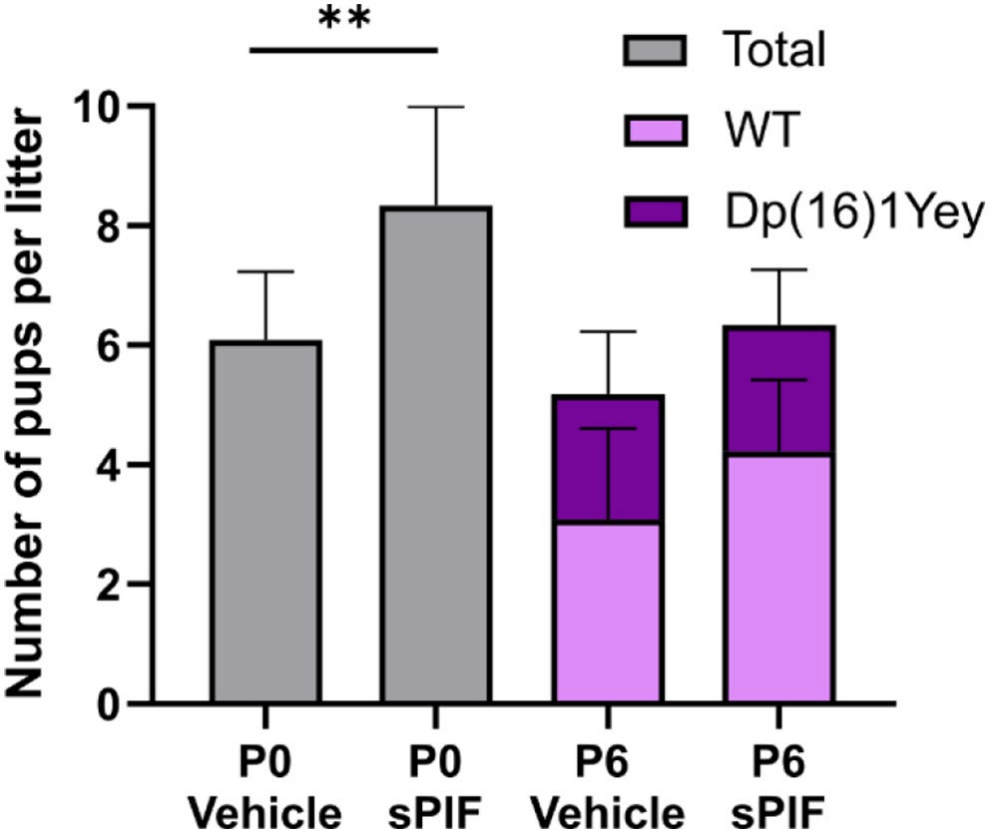
Treatment with sPIF does not affect the survival rate of Dp(16)1Yey pups. The pups in the litter received saline (*n* = 11 litters) or sPIF (*n* = 9 litters). Data are expressed as the mean ± SD and were analyzed in an unpaired, two-tailed Student’s t-test. ** *p* < 0.01

Li et al. has reported a survival rate of 38% for Dp(16)1Yey mice at weaning, with a normal Mendelian ratio of viable embryos at E18.5 [20]. We first investigated the effect of sPIF treatment in gestating females from embryonic day 14 until weaning of the pups on the survival rate. To that end, we administered sPIF or saline prenatally to females from E14 to P6. Treatment with sPIF was associated with a significantly greater number of pups per litter at birth (Fig. 1). In both groups, the survival rate of Dp(16)1Yey pups on P6 was the same as that reported by Li et al. (Fig. 1); this suggests that sPIF treatment does not worsen survival of pups.

### Treatment of Dp(16)1Yey pups with sPIF is associated with positive effects on markers of apoptosis, inflammation and neurogenesis and markers located on the mouse ortholog of human chromosome 21

Due to the expected positive effects of sPIF on apoptosis, we first evaluated the expression of genes involved in apoptosis on P6. The expression levels of the pro and anti-apoptotic genes, BCL2-associated X (*Bax*) and *Bcl2* were similar in WT pups and Dp(16)1Yey pups receiving vehicle (Table 1) but lower in sPIF-treated Dp(16)1Yey pups (Table 1). Transcription of the cellular inhibitor of apoptosis 2 (cIAP2) is induced by the nuclear factor-κB (NF-κB) pathway. cIAP2 mRNA expression was similar in nontreated Dp(16)1Yey mice and WT mice but was lower in sPIF-treated Dp(16)1Yey pups.

**Table 1:** Quantitative PCR analysis of mRNA extracted from whole brains of WT and Dp(16)1Yey pups on P6

mRNA level (%)	Vehicle-treated WT (*n* = 4)	sPIF-treated WT (*n* = 4)	Vehicle-treated Dp(16)1Yey (*n* = 4)	sPIF-treated Dp(16)1Yey (*n* = 4)
*Bax*	100 ± 4	54 ± 6^£^	93 ± 19	58 ± 5^$^
*Bcl2*	100 ± 4	53 ± 7^££^	90 ± 17	53 ± 7^$^
*cIPA2*	100 ± 5	72 ± 7^£^	94 ± 8	69 ± 10^$^
*Cyclin D1*	100 ± 6	83 ± 12	73 ± 4^*^	121 ± 3^$$^
*Dyrk1a*	100 ± 21	104 ± 24	183 ± 31^*^	116 ± 18^$^
*Gli2*	100 ± 17	56 ± 12^£^	65 ± 10^*^	120 ± 12^$^
*Iba1*	100 ± 25	101 ± 13	174 ± 23^*^	121 ± 22^$^
*IκBα*	100 ± 5	73 ± 5^£^	140 ± 13^***^	102 ± 4^$$^
*IL-6*	100 ± 9	67 ± 3^£^	147 ± 11^**^	108 ± 14^$^
*p65*	100 ± 6	75 ± 9	122 ± 5^*^	99 ± 10^$^
*S100B*	100 ± 10	78 ± 8^£^	155 ± 15^***^	110 ± 8^$^

Data are expressed as the mean ± SD and were analyzed in a two-way ANOVA followed by Fisher’s least squares difference test. ** *p* < 0.01 and *** *p* < 0.005 (sPIF-treated WT or vehicle-treated Dp(16)1Yey vs. vehicle-treated WT); ^$^*p* < 0.05 (sPIF-treated Dp(16)1Yey vs. vehicle-treated Dp(16)1Yey); ^£^*p* < 0.05 and ^££^*p* < 0.01 (sPIF-treated WT vs. vehicle-treated WT).

In view of the known beneficial effect of PIF treatment on neuroinflammation, we analyzed the effect of sPIF treatment on the expression of genes coding for proteins involved in the NF-κB pathway and of one of its target genes (interleukin (IL)-6). We found that sPIF treatment (i) was associated with a relative reduction in the elevated mRNA expression levels of inhibitor of nuclear factor kappa-b kinase subunit gamma (Ikbα), p65 and IL-6 usually observed in the brain of Dp(16)1Yey mice and (ii) had no negative effects on the brain of WT mice. We also analyzed the mRNA expression level of the microglial and macrophage marker ionized calcium-binding adapter molecule 1 (Iba1); the level was higher in nontreated Dp(16)1Yey mice than in WT mice, and sPIF-treatment restoring this increase.

**Fig. 2:**
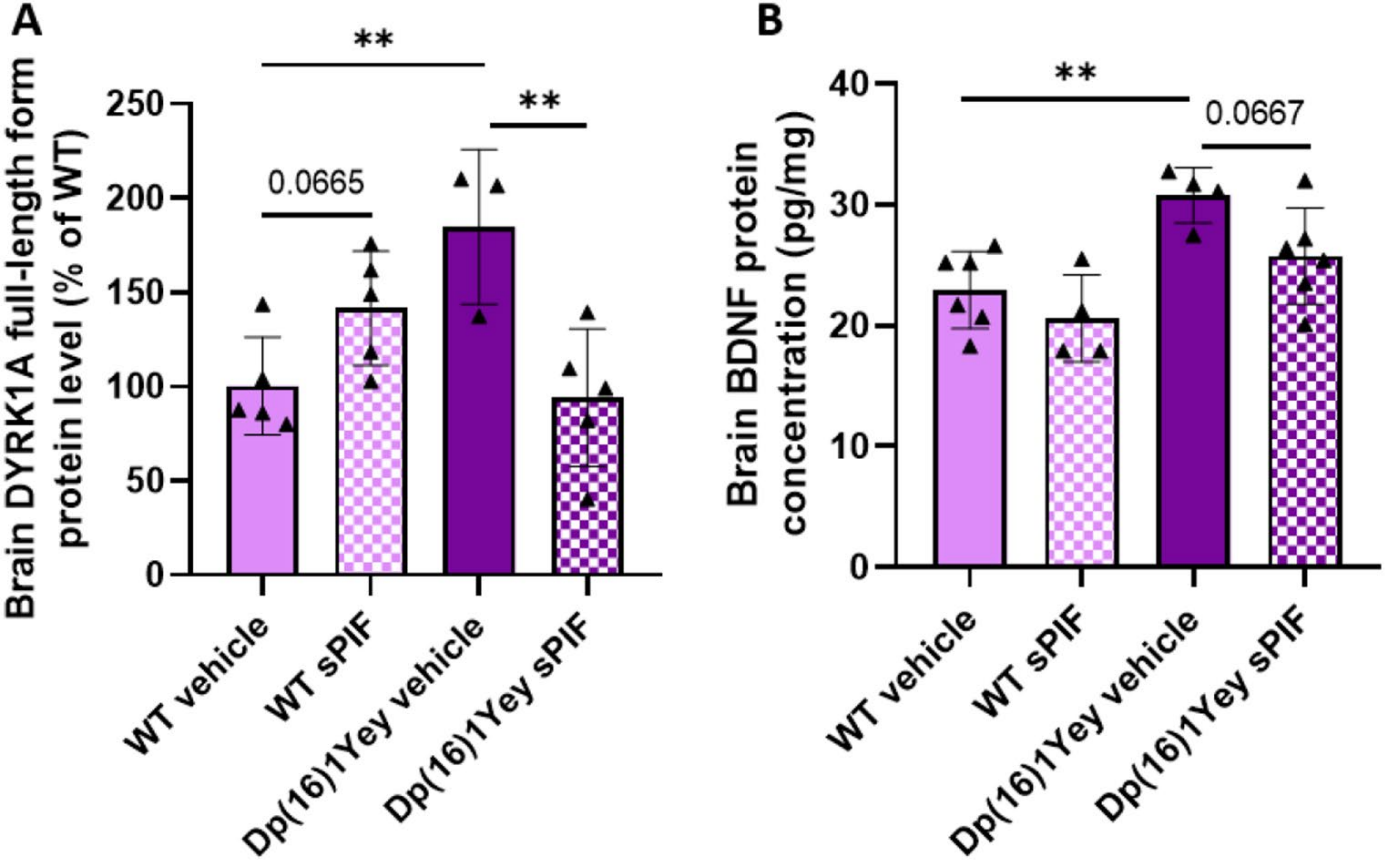
Treatment with sPIF restores DYRK1A and BDNF protein levels in the brain of Dp(16)1Yey pups on P6. Evaluation of the DYRK1A protein level in WT pups (vehicle: *n* = 5; sPIF: *n* = 5) and Dp(16)1Yey pups (vehicle: *n* = 3; sPIF: *n* = 5) (**A**). The brain BDNF protein concentration in WT pups (vehicle: *n* = 6; sPIF: *n* = 4) and Dp(16)1Yey pups (vehicle: *n* = 4; sPIF: *n* = 6) (**B**). Data are expressed as the mean ± SD and were analyzed in a two-way ANOVA followed by Fisher’s least squares difference test. ** *p* < 0.01

We also analyzed two markers located on the mouse orthologs of human chromosome 21 and being involved in cognitive impairments in people with DS. Dual specificity tyrosine-phosphorylation-regulated kinase 1 A *(Dyrk1A)* and S100 calcium binding protein β *(S100β)* were overexpressed in the brain of Dp(16)1Yey mice receiving vehicle but not in Dp(16)1Yey mice receiving sPIF (Table 1). The relative reduction on DYRK1A mRNA expression associated with sPIF treatment was also confirmed for expression of the corresponding protein (Fig. 2A).

We also investigated genes linked to neurogenesis. Cyclin D1 is involved in cell cycle regulation and is degraded via a ubiquitin-mediated proteolysis pathway at the end of the S-phase. Gli2 (a mediator of Sonic hedgehog (Shh) signaling pathway) is implicated in many aspects of growth and development. Low mRNA expression of cyclin D1 and glioma-associated oncogene family zinc finger 2 (Gli2) in Dp(16)1Yey mice was counteracted by sPIF treatment. However, sPIF treatment was associated with a lower Gli2 mRNA level in WT mice. We also analyzed BDNF, a neurotrophic factor known to be involved in neurogenesis. The brain concentration of BDNF protein was significantly higher in untreated Dp(16)1Yey pups than in WT pups, and sPIF treatment partially restoring its concentration (Fig. 2B).

### Treatment with sPIF is associated with low body weight in pups

We next observed the effects of sPIF treatment during the early postnatal period. First, we sought to determine whether the abnormally low body weight observed between P3 and P21 in Dp(16)1Yey pups could be countered by treatment with sPIF [24]. Treatment with sPIF was associated with significantly lower body weight in male and female WT pups on P5 and P11 (Table 2). The same association was found for female Dp(16)1Yey pups but not their male counterparts. To avoid possible bias from the influence of bodyweight on movements and strength, we chose to measure USVs and nest odor preference on P7 and P8, respectively.

**Table 2:** Treatment with sPIF is associated with low weight in male (M) and female (F) WT and Dp(16)1Yey pups on P5 and P11

Weight (g)		Vehicle-treated WT *n* = 42 M/34F	sPIF-treated WT *n* = 22 M/18F	Vehicle-treated Dp(16)1Yey *n* = 40 M/35F	sPIF-treated Dp(16)1Yey *n* = 16 M/20F
P5	Males	2.93 ± 0.10	2.45 ± 0.13^**^	2.82 ± 0.09	2.31 ± 0.09
	Females	3.18 ± 0.10	2.64 ± 0.14^**^	2.75 ± 0.09^**^	2.34 ± 0.10^$^
P11	Males	5.82 ± 0.15	4.77 ± 0.24^***^	5.64 ± 0.10	5.04 ± 0.25
	Females	6.20 ± 0.16	5.32 ± 0.20^**^	5.59 ± 0.11^**^	4.83 ± 0.30^$^

Data are expressed as the mean ± SD and were analyzed in a two-way ANOVA followed by Bonferroni’s post hoc test. ** *p* < 0.01 and *** *p* < 0.005 (sPIF-treated WT or vehicle-treated Dp(16)1Yey vs. vehicle-treated WT); $ *p* < 0.05 (sPIF-treated Dp(16)1Yey vs. vehicle-treated Dp(16)1Yey).

### Treatment with sPIF is associated with ameliorated communication in Dp(16)1Yey pups

**Fig. 3:**
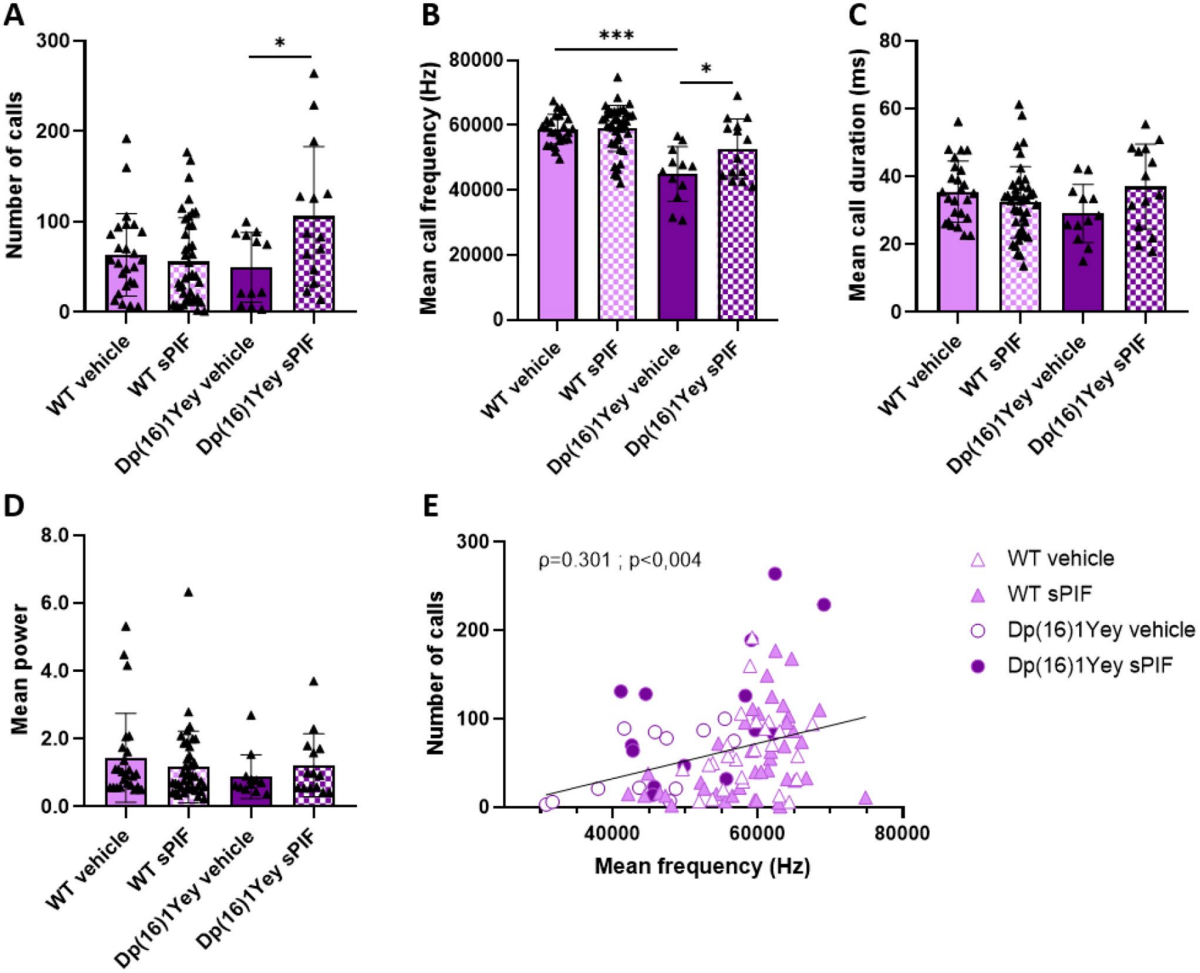
Treatment with sPIF restores the mean call frequency in Dp(16)1Yey pups. Evaluation of number of calls (**A**) mean call duration (**B**) mean frequency (**C**) and mean power (**D**) in WT (vehicle: *n* = 25; sPIF: *n* = 41) and Dp(16)1Yey (vehicle: *n* = 12; sPIF: *n* = 14). Data are expressed as the mean ± SD and were analyzed in a two-way ANOVA followed by Bonferroni’s post-hoc test. * *p* < 0.05 and *** *p* < 0.005. The correlation between the number of calls and the mean frequency was analyzed by calculating Pearson’s correlation coefficient (**E**)

We did not observe a difference between male and female pups in the USVs measurements, and so we pooled the data from each sex (Fig. 3). The numbers of calls were similar in Dp(16)1Yey and WT pups but were higher in sPIF-treated Dp(16)1Yey pups than in untreated Dp(16)1Yey pups (Fig. 3A). The mean call frequency was significantly lower in Dp(16)1Yey pups than in WT pups but not in sPIF-treated Dp(16)1Yey pups (Fig. 3B). The duration and power of the calls were not significantly related to the genotype or treatment (Fig. 3C and D). Lastly, the number of calls was positively correlated with the mean call frequency (Fig. 3E).

### Treatment with sPIF is associated with ameliorated social behavior in Dp(16)1Yey pups

**Fig. 4:**
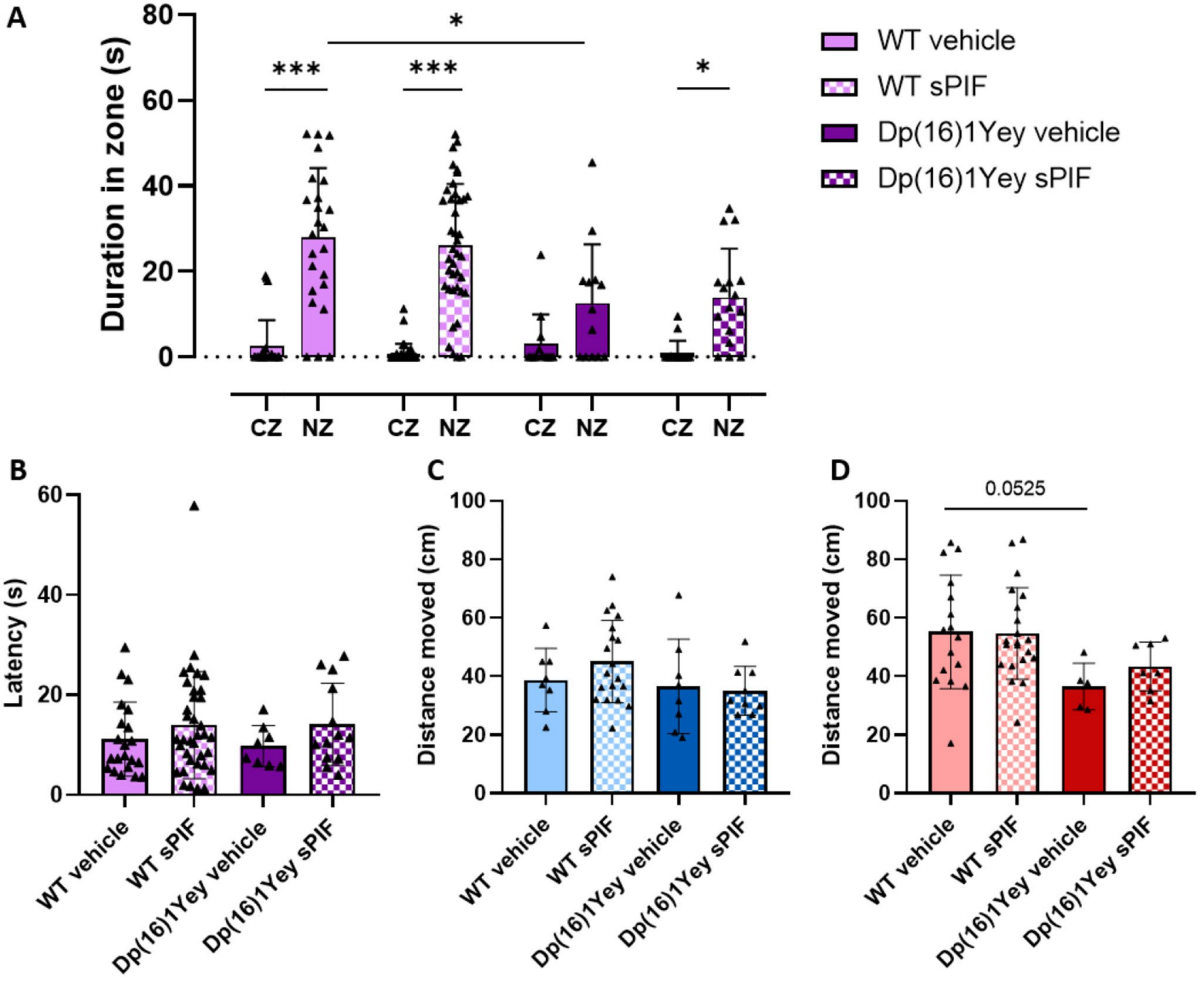
Treatment with sPIF ameliorates juvenile social behavior in Dp(16)1Yey pups. The results are presented as the mean time spent in the clean zone (CZ) and the nest zone (NZ) (**A**) and the time taken to enter in NZ (**A**) by WT pups (vehicle: *n* = 24; sPIF: *n* = 40) and Dp(16)1Yey pups (vehicle: *n* = 13; sPIF: *n* = 16). Data are expressed as the mean ± SD and were analyzed in a two-way ANOVA followed by Bonferroni’s post-hoc test. Due to a sex difference, locomotor activity was evaluated as the distance moved by males (vehicle-treated WT: *n* = 8; sPIF-treated WT: *n* = 19; vehicle-treated Dp(16)1Yey: *n* = 8; sPIF-treated Dp(16)1Yey: *n* = 9) (**A**) and females (vehicle-treated WT: *n* = 16; sPIF-treated WT: *n* = 21; vehicle-treated Dp(16)1Yey: *n* = 5; sPIF-treated Dp(16)1Yey: *n* = 7) separately (**A**). Data are expressed as the mean ± SD and were analyzed in a two-way ANOVA followed by Fisher’s least squares difference test. * *p* < 0.05 and *** *p* < 0.005

The nest odor preference test was performed on P8. There was no male vs. female differences in the time spent in the zones and the time taken to reach the NZ. So, both sexes were gathered. All the pups except vehicle-treated Dp(16)1Yey pups spent significantly more time in the NZ than in the CZ (Fig. 4A). Moreover, Dp(16)1Yey pups significantly spent less time in the NZ than WT pups did, and sPIF treatment was associated with spending a greater proportion of time in the NZ. There were no intergroup differences in the time before first entering the NZ (Fig. 4B). The proportion of pups entering the NZ was 61.5% for vehicle-treated Dp(16)1Yey pups, 87.5% for WT pups, 100% for sPIF-treated female Dp(16)1Yey pups, and 66.6% for sPIF-treated male Dp(16)1Yey pups (Table 3). The distance moved was measured as a guide to locomotor activity, (Fig. 4C and D). Vehicle-treated Dp(16)1Yey female pups moved less (albeit not significantly) than WT pups. However, sPIF treatment did not restore this impairment in locomotor activity.

**Table 3:** Treatment with sPIF is associated with greater homing to the nest zone (NZ) by female Dp(16)1Yey pups

		Vehicle-treated WT	sPIF-treated WT	Vehicle-treated Dp(16)1Yey	sPIF-treated Dp(16)1Yey
Female pups	Total tested	16	21	5	7
	Number reaching the NZ	15	18	3	7
	Proportion	**93.8%**	**85.7%**	**60%**	**100%**
Male pups	Total tested	8	19	8	9
	Number reaching the NZ	6	18	5	6
	Proportion	**75%**	**94.7%**	**62.5%**	**66.6%**

### Treatment with sPIF ameliorates neurogenesis and does not affect microglia and astrocytes in Dp(16)1Yey pups

**Fig. 5:**
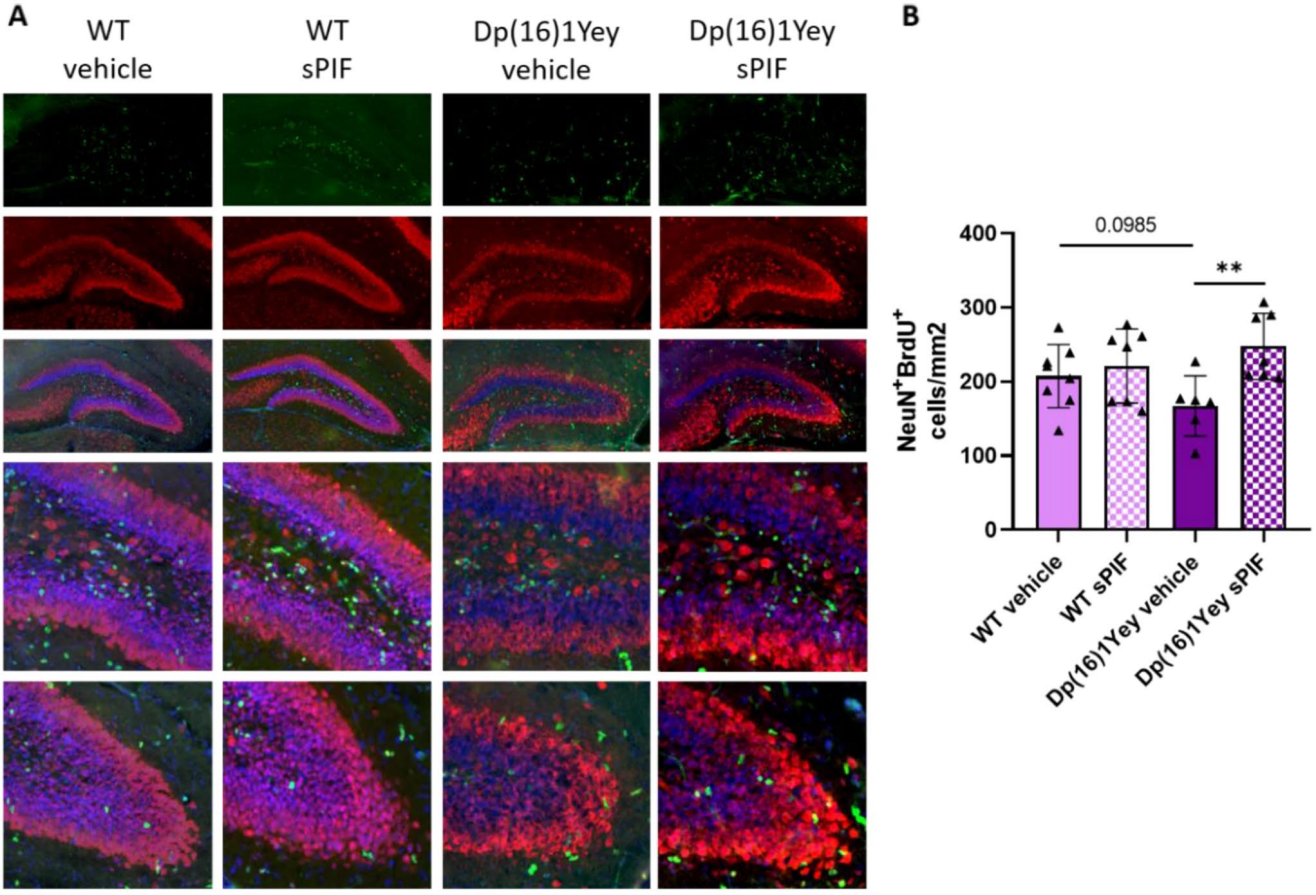
Treatment with sPIF restores neurogenesis in Dp(16)1Yey mice pups. Representative images of immunostaining of NeuN (red) and BrdU (green) in the dentate gyrus of WT pups (vehicle: *n* = 8; sPIF: *n* = 7) and Dp(16)1Yey pups (vehicle *n* = 6; sPIF: *n* = 7) (**A**). Quantification of the number of NeuN-and BrdU-positive cells in the dentate gyrus, normalized against the surface area (**A**). Data are expressed as the mean ± SD and were analyzed in a two-way ANOVA. An interaction between genotype and treatment was not found, and pairs of groups were compared in Student’s t-test. ** *p* < 0.01

In view of sPIF’s effects on neuronal protection, we used histologic technique to examine early postnatal hippocampal neurogenesis. Mice received BrdU on P10 (24 h before sacrifice), to label proliferating cells. Numbers of BrdU- and neuronal nuclei (NeuN)- positive cells in the dentate gyrus were counted (Fig. 5A). The level of neurogenesis was lower in Dp(16)1Yey pups than in WT pups, although the intergroup difference was not statistically significant. However, the level of neurogenesis was significantly higher in sPIF-treated Dp(16)1Yey pups than in untreated Dp(16)1Yey pups (Fig. 5B).

It is known that sPIF interacts with microglia and reduces the activation of this cell type [17]. A significantly lower microglia count was observed in the somatosensory cortex and the dentate gyrus of the Dp(16)1Yey pups, relative to WT pups (Fig. S1B, S1E). The branches of microglial cells in the somatosensory cortex were shorter in Dp(16)1Yey pups than in WT pups (Fig. S1C, S1F); this shortening is suggestive of an amoeboid cell shape and an activated cell state and is concordant with the elevated expression of Iba1 observed on P6 (Table 1). This phenotype was not found in the dentate gyrus. Treatment with sPIF did not appear to have an effect on Dp(16)1Yey pups but did influence the activated state in the somatosensory cortex in WT pups. There were no significant intergroup differences in the number of BrdU-positive cells and Iba1 positive cells in the dentate gyrus (Fig. S1D).

We also assessed the numbers of astrocytes and their proliferation; there were no differences between WT and Dp(16)1Yey mice in these variables in the dentate gyrus or somatosensory cortex (Fig. S2). Treatment with sPIF did not have an effect other than on the astrocyte count in the somatosensory cortex of WT pups.

### Treatment with sPIF restores neurogenesis through the regulation of cyclin D1 and c-myc

**Fig. 6:**
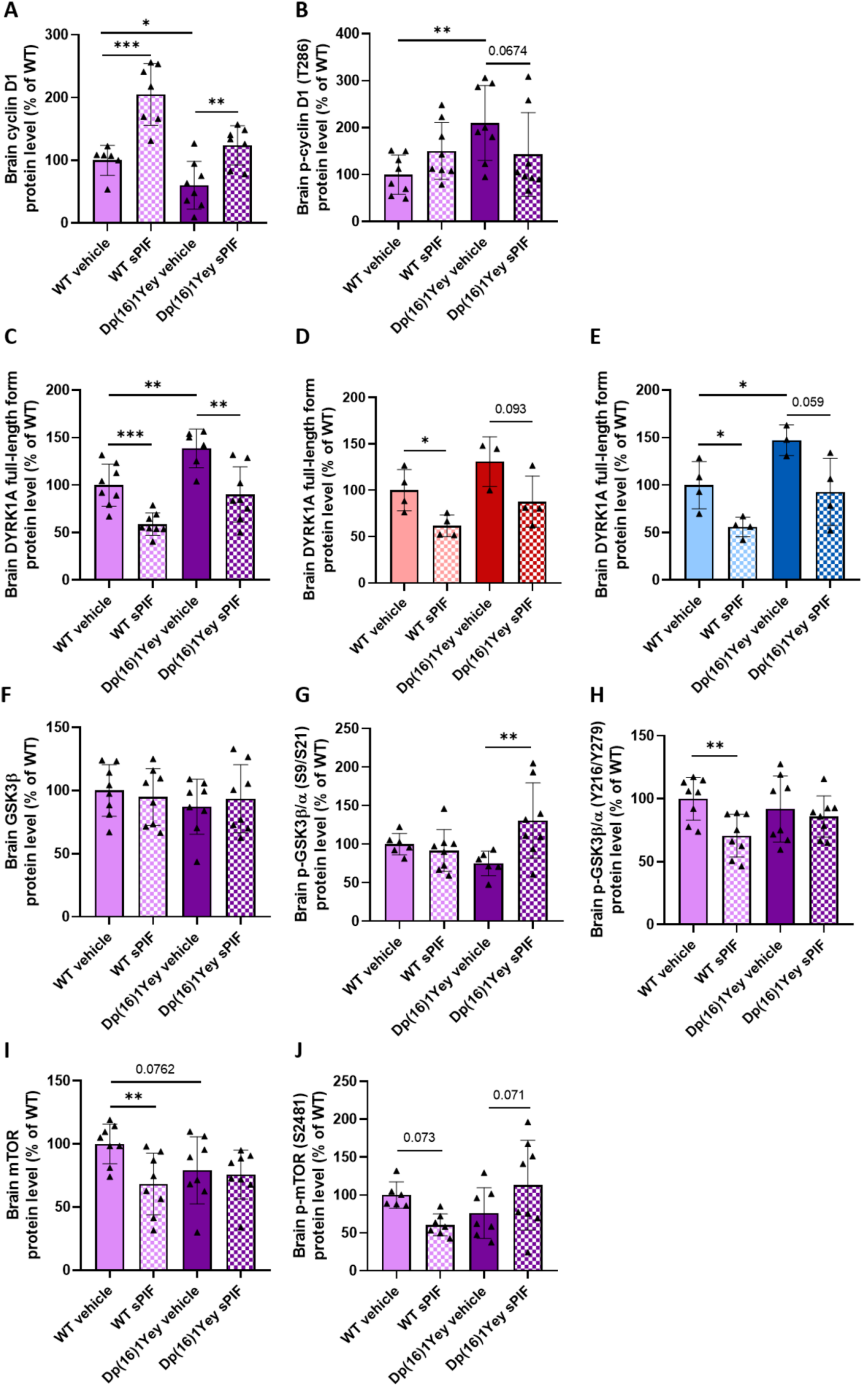
Treatment with sPIF restores the level of cyclin D1 phosphorylation at threonine 286 (T286) through regulation of the DYRK1A protein level and the GSK3 pathway on P11. Evaluation of cyclin D1 protein level (**A**) and its phosphorylation at T286 (T286) (**B**) in WT pups (vehicle: *n* = 6/8; sPIF: *n* = 8/7) and Dp(16)1Yey pups (vehicle: *n* = 8/8; sPIF: *n* = 8/7); the DYRK1A protein level in WT pups (vehicle: *n* = 8; sPIF: *n* = 8) and Dp(16)1Yey pups (vehicle: *n* = 8; sPIF: *n* = 8) (**C**), results for female (in red) (**D**) and male (in blue) (**E**) being presented in WT pups (vehicle: *n* = 4 F/4 M; sPIF: *n* = 4 F/4 M) and Dp(16)1Yey pups (vehicle: *n* = 4 F/4 M; sPIF: *n* = 4 F/4 M); the GSK3β protein level (**F**) and the level of GSK3β/α phosphorylation at S9/S21 (**G**) and at Y216/Y279 (**H**) in WT pups (vehicle: *n* = 8/7/8; sPIF: *n* = 8/8/8) and Dp(16)1Yey pups (vehicle: *n* = 8/6/8; sPIF: *n* = 8/8/8); the mTOR protein level (**I**) and level of phosphorylation at S2481 (**J**) in WT pups (vehicle: *n* = 8/6; sPIF: *n* = 8/7) and Dp(16)1Yey pups (vehicle: *n* = 8/7; sPIF: *n* = 8/8). Data are expressed as the mean ± SD and were analyzed in a two-way ANOVA followed by Fisher’s least squares difference test or an unpaired t-test. * *p* < 0.05; ** *p* < 0.01; *** *p* < 0.005

In experiments on Dp(16)1Yey mice on P11, we investigated a number of molecular mechanisms related to sPIF’s effect on neurogenesis. Cyclin D1 is a cell cycle regulator and controls progression through the G1 phase and the G1/S transition. In view of our results for DYRK1A mRNA and protein expression on P6 (Table 1; Fig. 2), we explored the phosphorylation status of cyclin D1 threonine 286 (T286); phosphorylation of this residue is known to be induced by DYRK1A and glycogen synthase kinase 3 (GSK3) β, a target of DYRK1A. The level of cyclin D1 protein on P11 was lower in untreated Dp(16)1Yey mice than in WT pups, whereas sPIF treatment of Dp(16)1Yey mice pups restored this variable to WT levels (Fig. 6A). Phosphorylation of cyclin D1 mediates its ubiquitination and degradation. The effect of sPIF treatment on T286 phosphorylation is consistent with the effect on cyclin D1 level (Fig. 6B). As observed on P6, the low brain DYRK1A protein level on P11 was restored by sPIF treatment of the Dp(16)1Yey mice (Fig. 6C, D, E). There were no intergroup differences in the GSK3β protein level (Fig. 6F). The activity of GSK3β/α is inhibited by phosphorylation on serines 9/21 and activated by phosphorylation on tyrosines 216/279. The phosphorylation levels were similar in WT mice and untreated Dp(16)1Yey mice. However, the level of serine phosphorylation was greater in sPIF-treated Dp(16)1Yey mice (Fig. 6G and H), which suggested a decrease in GSK3 activity. This effect might be due to an increase in mammalian target of rapamycin (mTOR) activity, which is known to inhibit GSK3β. The level of mTOR serine 2481 phosphorylation was higher in sPIF-treated Dp(16)1Yey mice than in untreated Dp(16)1Yey mice, which might reflect mTOR’s activity (Fig. 6I and J).

**Fig. 7:**
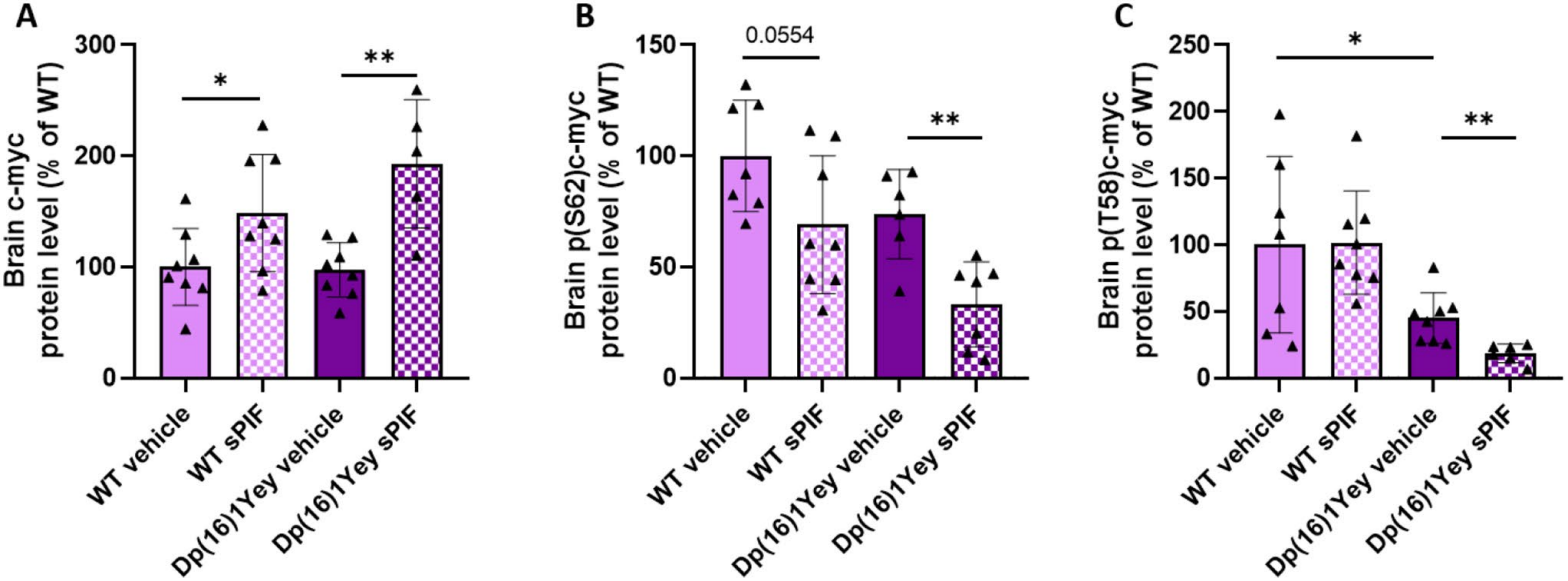
Treatment with PIF is associated with a higher level of c-myc protein level via regulation of phosphorylation at threonine 58 (T58) and serine 62 (S62). Evaluation of the c-myc protein level in WT pups (vehicle: *n* = 8; sPIF: *n* = 8) and Dp(16)1Yey pups (vehicle: *n* = 8; sPIF: *n* = 5) (**A**), the level of phosphorylation at S62 (**B**) and T58 (**C**) in WT pups (vehicle: *n* = 7/7; sPIF: *n* = 8/8) and Dp(16)1Yey pups (vehicle: *n* = 6/8; sPIF: *n* = 7/6). Data are expressed as the mean ± SD and were analyzed in a two-way ANOVA followed by unpaired t-test in pair-wised groups. * *p* < 0.05; ** *p* < 0.01

The transcription factor c-myc is also a target of DYRK1A and GSK3β and is involved in cell cycle regulation. GSK3β and DYRK1A induce the phosphorylation of c-myc on threonine 58 (T58) and serine 62 (S62), respectively, which leads to c-myc’s ubiquitination and degradation. The level of c-myc was similar in WT mice and Dp(16)1Yey mice but the level of T58 phosphorylation was lower in the Dp(16)1Yey mice. Treatment with sPIF was associated with higher protein levels of c-myc in both WT and Dp(16)1Yey pups (Fig. 7A), relative to untreated pups. This observation was consistent with lower levels of phosphorylation at S62 (Fig. 7B) in WT and Dp(16)1Yey pups and at T58 (Fig. 7C) in Dp(16)1Yey pups.

### Treatment with sPIF partially restores the working memory impairment in female adult Dp(16)1Yey mice

**Fig. 8:**
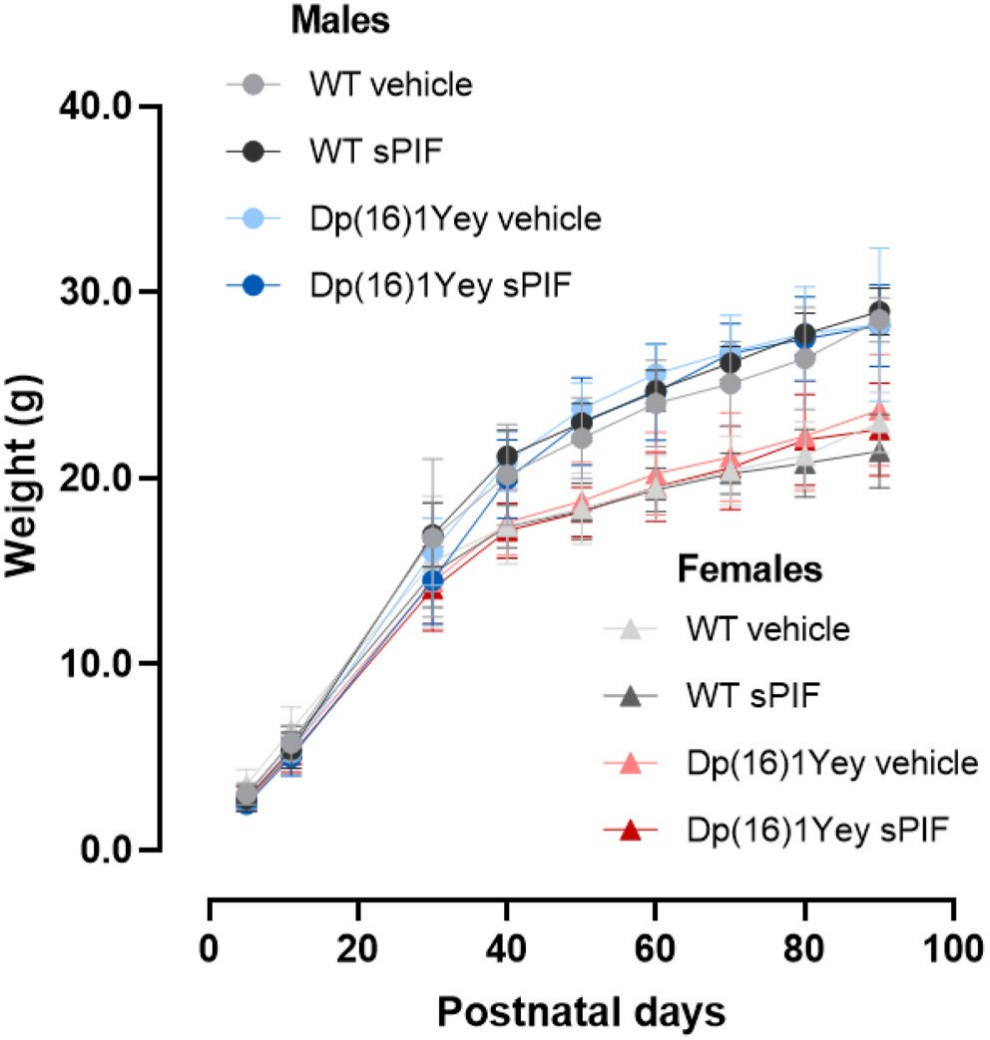
Treatment with sPIF is not associated with lower body weight in adult mice. Weights of male and female adult WT mice (vehicle: *n* = 8 M/8F; sPIF *n* = 14 M/8F) and adult Dp(16)1Yey mice (vehicle: *n* = 7 M/9F; sPIF: *n* = 10 M/8F) mice. Data are expressed as the mean ± SD and were analyzed in a two-way ANOVA followed by Fisher’s least squares difference test

We sought to determine whether the administration of sPIF during the prenatal and perinatal period influenced adult mice. We first assessed the mice’s bodyweight up to P90. Males were heavier than females (Fig. 8). Regardless of the type of treatment, adult WT and Dp(16)1Yey mice did not differ significantly with regard to bodyweight; hence, the weight difference for juveniles was no longer present in adulthood. However, adult Dp(16)1Yey mice, either male or female, shows reduced brain weight (Table 4). sPIF treatment significantly restores the brain weight in the Dp(16)1Yey male and female mice (Table 4).

**Table 4:** Treatment with sPIF restores brain weight in male (M) and female (F) adult Dp(16)1Yey mice

Brain weight/total weight (% of WT)		Vehicle-treated WT *n* = 18 M/8F	sPIF-treated WT *n* = 14 M/8F	Vehicle-treated Dp(16)1Yey *n* = 8 M/9F	sPIF-treated Dp(16)1Yey *n* = 10 M/8F
adult	Males	101 ± 1	98 ± 1	95 ± 1*	101 ± 2^$^
	Females	101 ± 2	106 ± 2	91 ± 3^**^	99 ± 3^$^

Data are expressed as the mean ± SD and were analyzed in a two-way ANOVA followed by Bonferroni’s post hoc test. * *p* < 0.07, and **p < 0.05 (sPIF-treated WT or vehicle-treated Dp(16)1Yey vs. vehicle-treated WT); $ *p* < 0.05 (sPIF-treated Dp(16)1Yey vs. vehicle-treated Dp(16)1Yey).

**Fig. 9:**
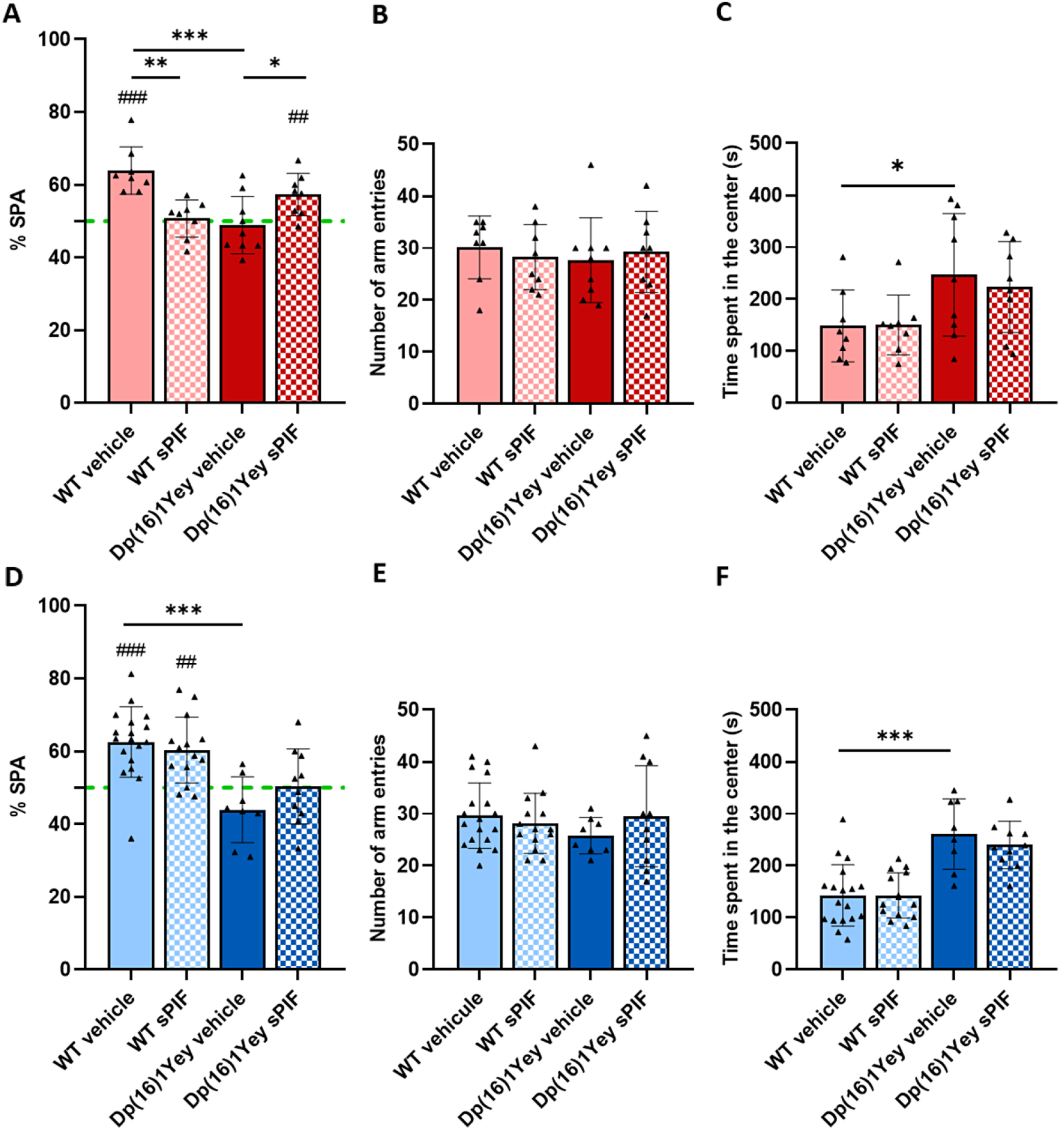
Treatment with sPIF partially restores SPA in Dp(16)1Yey female adult mice. Results for male (in blue) and female (in red) are presented as the proportion of SPAs (%) (**A**, **D**), the number of arm entries (**B**, **E**), and time spent in the center of the maze (**C**, **F**) in WT mice (vehicle: *n* = 18 M/8F; sPIF: *n* = 14 M/8F) and Dp(16)1Yey mice (vehicle: *n* = 8 M/9F; sPIF: *n* = 10 M/8F). The green hashed line represents the theoretical value for random alternation between arms. Data are expressed as the mean ± SD. For the SPA in females, data were analyzed in a two-way ANOVA followed by Fisher’s least squares difference test. For the other analysis, no interactions were found between genotype and treatment and pairs of groups were analyzed in unpaired two-tailed Student’s t-test. * *p* < 0.05, ** *p* < 0.01, *** *p* < 0.005. The mean proportion of SPAs of each group was compared with the value of 50% in a one-sample t-test. ## *p* < 0.01, ### *p* < 0.005

We next used a Y-maze to study working memory. Given that performance differences between male and female mice were observed, each sex was studied separately (Fig. 9). In male and female WT mice, the SPA rate was over 60%, i.e. greater than the value of 50% corresponding to random alternation between arms (Fig. 9A and D). As described previously, the SPA rate was significantly lower in Dp(16)1Yey mice than in WT mice. Treatment with sPIF rescues performance in female Dp(16)1Yey mice (Fig. 9A), the difference for male not being statistical (Fig. 9D). Locomotor activity (evaluated by the number of arm entries) did not appear to be influenced by the genotype or the treatment (Fig. 9B and E). Lastly, Dp(16)1Yey mice (whether treated with sPIF or vehicle) spent significantly more time in the center of the maze than WT mice. This result, associated with the groups’ similar numbers of arm entries, suggested that Dp(16)1Yey mice explored the arms less than WT mice did (Fig. 9C and F).

## Discussion

The results of our study of the Dp(16)1Yey mouse (a mouse model of DS) indicate that sPIF treatment has beneficial effects during the prenatal and perinatal periods. sPIF rescues the impairment in postnatal hippocampal neurogenesis - possibly through the factor’s effects on the cell cycle and/or pathways that promote neuronal growth. Treatment with sPIF did not worsen the survival of Dp(16)1Yey pups. Furthermore, treatment with sPIF enhanced the Dp(16)1Yey pups’ social communication in response to maternal separation and partially rescued the impairment in hippocampal-dependent working memory in adult Dp(16)1Yey mice.

We found that Dp(16)1Yey mice have a lower bodyweight (relative to WT pups) from P5 onwards. These results are in line with those reported by Goodliffe et al. from P5 to P21, with significant differences between Dp(16)1Yey pups and WT pups and between male and female Dp(16)1Yey pups [24]. Conceivably, the Dp(16)1Yey pups’ weight difference might affect the acquisition of reflexes. We therefore chose to measure the pups’ behavioral and social responses on P7 and P8 (before eye opening), when the weight difference is smaller. Our results suggest that early social communication is impaired in Dp(16)1Yey pups, previously demonstrated at 5 months of age [25]. This impairment might be related to neurodevelopmental disorders [26], although we cannot rule out an influence of poor olfaction or low locomotor activity. Researchers have observed an impairment in olfaction and a low neuron count in the olfactory bulb in the adult Ts65Dn mouse model of DS [27, 28]. These impairments are not apparent on P15 and indicate the presence of an age-dependent process, as observed in people with DS [29]. Female Dp(16)1Yey mice covered less distance during the test (albeit not significantly), which might be a consequence of the lower weight. Guedj et al. reported that 8-day-old Ts1Cje mice increased their USVs after being separated from their mother for 20 min or in response to a temperature of 16 °C [30]. The maternal preference test on P12 showed that the pups took longer to reach the maternal zone. The peak in USVs occurs later in Ts65Dn pups than in controls, and Ts65Dn pups take longer to reach the maternal zone [31]. A new mouse model of DS (Ts66Yah) has been derived from the Ts65Dn model. Ts66Yah mice have the same gene duplication as Ts65Dn mice, except that genes on chromosome 17 (not related to human chromosome 21) have been removed using the CRISPR/Cas9 system [32]. Duchon et al. found that relative to control pups, male Ts66Yah pups (whose trisomy originates from the mother) had a greater total number of USVs and a lower proportion of short vocalizations. Female Ts66Yah pups emit a significantly higher number of vocalizations, regardless of the origin of the duplication. In the maternal preference test, these mice took slightly (but not significantly) longer to reach the maternal zone and spent less time there [33]. Treatment with sPIF was associated with better performance by Dp(16)1Yey pups in tests evaluating the response to maternal separation. It is known that sPIF has neuroprotective effects in models characterized by neuronal loss [16, 17, 34]. The restoration of postnatal hippocampal neurogenesis might correct these cognitive impairments.

Histological analyses on P11 showed a slightly (but not significantly) lower level of hippocampal neurogenesis in Dp(16)1Yey pups. Goodliffe et al. reported that the Dp(16)1Yey model did not exhibit defects in brain development and neurogenesis at the embryonic stage [24]. The researchers suggested that the acquisition delays observed during the first 21 days of life in Dp(16)1Yey were related to mild anomalies during the embryonic period or the onset of impairments during the early postnatal period. In contrast to other treatments tested in mouse models of DS, sPIF had a positive effect on neurogenesis in Dp(16)1Yey pups. For example, the administration of fluoxetine from P3 to P15 in Ts65Dn mice restored neural proliferation in the dentate gyrus, striatum, subventricular zone, and neocortex (as measured on P15) [35]. Prenatal administration of fluoxetine also had these beneficial effects [36] and restored cognitive performance in general and hippocampal-dependent functions in particular. Adult Dp(16)1Yey mice and WT controls have similar weight curves, and so we chose to use a working memory test (the Y-maze); the SPA rate was lower in Dp(16)1Yey mice [22, 37]. Similarly, Dp(16)1Yey mice have normal levels of locomotor activity but spent more time in the center [38, 39]. sPIF treatment partially restored the level of cognitive performances and had a long-term effect.

We studied the expression of cyclin D1 (involved in neurogenesis and neuronal differentiation through cell cycle regulation during the G1 phase [40, 41]) and the involvement of the GSK3/mTOR pathway. We observed low cyclin D1 expression in Dp(16)1Yey mice, which might lead to quiescence in phase G1 of the cell cycle. Cyclin D1 is known to be regulated by DYRK1A, which induced rapid degradation of the cyclin by phosphorylation at T286 [40]. Molecular analyses on P6 and P11 showed that sPIF treatment was associated with the normalization of DYRK1A and cyclin D1 levels, low GSK3 activity, and elevated mTOR activity. Interestingly, a discrepancy between P6 and P11 in DYRK1A protein level was found in WT mice treated by sPIF. Recent study demonstrated the variation of DYRK1A expression especially with age, more precisely between P6 and P15 in both sexes. These data could explain our results obtained in WT mice between P6 and P11 [42]. Moreover, based on the mental retardation autosomal dominant 7 syndrome characterized by DYRK1A haploinsufficiency [43], the decreased DYRK1A protein level by 45% at P11 in female and male mice and the lower SPA rate in female WT mice after sPIF prenatal treatment suggest the need to adjust the dose based on gender.

Overexpression of GLI2 can enhance cyclin D1 expression [41]. The transcription factor GLI2 is activated by the Shh pathway, which is known to be involved in neurogenesis [44]. Low GLI2 expression has been observed in neuronal precursors derived from induced pluripotent stem cells obtained from people with DS [45]. We also observed low GLI2 expression in the Dp(16)1Yey mouse, which suggests that Shh signaling is impaired in this model of DS. Treatment of Dp(16)1Yey mice with sPIF restored levels of cyclin D1 and GLI2.

Our network analysis of DYRK1A’s interactome revealed an indirect interaction with c-myc [46], which is important during neurogenesis [47]. In line with the effect on sPIF treatment on GSK3 and DYRK1A expression, we observed low c-myc phosphorylation level and an elevated c-myc level in Dp(16)1Yey mice; our results demonstrate the importance of this pathway in neurogenesis.

Dendritic morphology and synaptic plasticity are impaired in DS and are critically influenced by BDNF. We observed an elevated level of BDNF in the brains of Dp(16)1Yey mice. Adult Ts1Cje mice also exhibit abnormally high hippocampal BDNF levels [48]. People with DS have elevated plasma levels of BDNF [49]. Given that BDNF readily crosses the blood–brain barrier [50], this elevation probably reflects a higher level of BDNF in the brain. Treatment with sPIF restored BDNF levels in Dp(16)1Yey mice; this might reflect a systemic action.

We observed a low number of microglia in the dentate gyrus and somatosensory cortex in Dp(16)1Yey pups. On the molecular level, Dp(16)1Yey mice overexpressed Iba1 in the brain on P6. These results are consistent with the microglial activation (observed by immunofluorescence) in the somatosensory cortex on P11. Furthermore, we did not observe differences in microglial morphology (process length) in the dentate gyrus. Pinto et al.‘s study of Dp(16)1Yey mice on P22 showed that the number of hippocampal microglial cells was similar to that found in control mice [51]. These researchers also reported changes in microglial morphology in the hippocampus (indicating activation) and low dendritic spine density. The protocol used in Pinto et al.‘s study was based on weaning pups on P16 and analyzing the microglia on P22. Previous studies have shown that maternal separation induces stress and neuroinflammation in pups [52]. In our study, the pups were not separated from their mother on P11. It is possible that in Pinto et al.‘s study, weaning on P16 led to exacerbated neuroinflammation in the pups. Even though sPIF treatment restored the expression of genes involved in inflammation linked to the NF-κB pathway (p65, IL-6, Iκb, and Iba1), it had no effect was on microglial activation in the somatosensory cortex; this contrasts with the effects on treatment with PLX3397 [51]. Several studies have shown that sPIF is associated with a lower number of Iba1-positive cells [34, 53]. In a placental cell line, sPIF reduced the lipopolysaccharide-induced expression of IL-6 [53]. However, these studies focused on acute neuroinflammation, rather chronic neuroinflammation in which the number of microglial cells is higher.

We observed the elevated expression of S100β in Dp(16)1Yey pups, even though its gene is only present in two copies, suggesting epistatic interactions between duplicated genes in Dp(16)1Yey mice and S100β gene. This result has been previously found in the cerebellum of Ts1Cje mice [54]. This elevation was normalized by sPIF treatment. S100β is synthesized by astrocytes and is considered to be a marker of neuron damage [55]. However, control WT pups and Dp(16)1Yey did not differ significantly with regard to the number and proliferation of S100β-positive astrocytes, suggesting no real involvement of increased S100 β gene expression in Dp(16)1Yey mice.

## Conclusions

Taken as a whole, our results suggest that sPIF treatment had a neuroprotective effect (rather than an anti-inflammatory effect) in Dp(16)1Yey mice. We identify several novel neuroprotective properties of the PIF peptide. Therapeutic strategies based on reducing the activity of DYRK1A (e.g. leucettine L41 and a green tea extract enriched in epigallocatechin gallate) have shown beneficial effects in DS models, including Dp(16)1Yey mice [38, 56, 57]. Importantly, sPIF treatment influences the level of DYRK1A protein. Although we did not analyze the sPIF effect on DYRK1A activity, its positive effect in reducing DYRK1A protein level in Dp(16)1Yey mice show a better efficacy than conventional type I ATP-competitive inhibitors which do not decrease DYRK1A protein level. The fact that a low DYRK1A protein level was observed following the administration of another peptide [58] demonstrates the promise of peptide therapy. When combined with the literature data, our present results further strengthen the hypothesis whereby the cognitive dysfunction linked to gene deregulation can be corrected, adjustment of DYRK1A being one of the explanations for the positive behavioral effects demonstrated. Although the underlying mechanism remains to be characterized in adult mice, our findings might guide future research on successful PIF therapy.

### Electronic supplementary material

Below is the link to the electronic supplementary material.

Supplementary Material 1

## Data Availability

The datasets generated and analyzed during the current study are available from the corresponding authors on reasonable request.

## Abbreviations

Bax: BCL2-associated X
BDNF: brain-derived neurotrophic factor
cIAP2: cellular inhibitor of apoptosis 2
DS: Down syndrome
DYRK1A: dual specificity tyrosine-phosphorylation-regulated kinase 1 A
Gli2: glioma-associated oncogene family zinc finger 2
GSK3: glycogen synthase kinase 3
Iba1: ionized calcium-binding adapter molecule 1
Ikbα: inhibitor of nuclear factor kappa-b kinase subunit gamma
IL: interleukin
mTOR: mammalian target of rapamycin
nest zone: NZ
NeuN: neuronal nuclei
NF-κB: nuclear factor-κB
PIF: PreImplantation Factor
S100b: S100 calcium binding protein b
Shh: Sonic hedgehog
SPA: spontaneous alternation
sPIF: synthetic PreImplantation Factor
USVs: ultrasonic vocalizations
