# Supplementary material for: Prenatal treatment with preimplantation factor improves early postnatal neurogenesis and cognitive impairments in a mouse model of Down syndrome
Source: Cell Mol Life Sci. 2024 May 13;81(1):215. doi: 10.1007/s00018-024-05245-9 (PMC11090972; doi:10.1007/s00018-024-05245-9)
Supplement: Supplementary file 1 — Supplementary Material 1 [file 18_2024_5245_MOESM1_ESM.docx]

### **Supplementary Information**

**Table S1** **Primers used for real-time quantitative RT-PCR of specific mRNAs.**


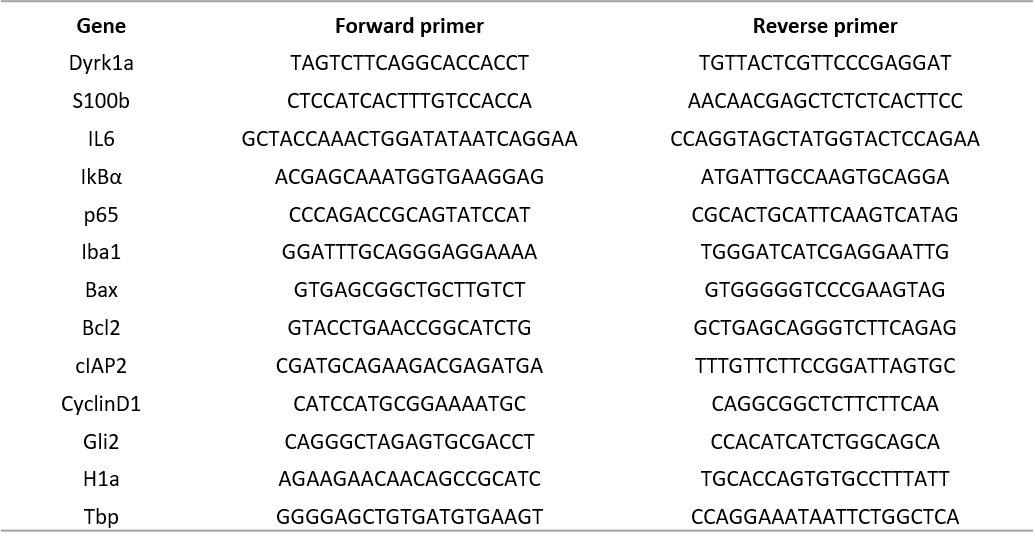


**Fig S1 sPIF has no effect on microglia in Dp(16)1Yey mice pups.** Representative images of immunostaining of Iba1 (red) of WT pups (vehicle: n=7; sPIF: n=6) and Dp(16)1Yey pups (vehicle n=6; sPIF: n=6) in the dentate gyrus and somatosensory cortex (A). Quantification of the number of Iba1-positive cells (normalized against the surface area) in the dentate gyrus (B) and the somatosensory cortex (E). Quantification of the length of branches in the dentate gyrus (C) and in the somatosensory cortex (F). Quantification of the number of Iba1- and BrdU-positive cells (normalized against the surface area) in the dentate gyrus in WT pups (vehicle: n=5; sPIF: n=6) and Dp(16)1Yey pups (vehicle n=6; sPIF: n=6) (D). *n* = 51 to 370 microglia were counted per group. Data are expressed as the mean ± SD and were analyzed in a two-way ANOVA followed by Fisher’s least squares difference test. For Iba1-positive cells in dentate gyrus, there was no interaction between the genotype and treatment and pairs of groups were analyzed in an unpaired, two-tailed Student’s t-test. * p<0.05; ** p<0.01; *** p<0.005

**Fig. S2 Treatment with sPIF had no effect on the number and proliferation of astrocytes in Dp(16)1Yey pups.** Quantification of the number of S100β-positive cells (normalized against the surface area) in the dentate gyrus (A) and the somatosensory cortex (C) in WT pups (vehicle: n=6; sPIF: n=6) and Dp(16)1Yey pups (vehicle n=6; sPIF: n=6). Quantification of the number of S100β- and BrdU-positive cells in the dentate gyrus (normalized against the surface area) in WT pups (vehicle: n=7; sPIF: n=5) and Dp(16)1Yey pups (vehicle n=6; sPIF: n=7) (B). The data are expressed as the mean ± SD and were analyzed in a two-way ANOVA. For S100β-positive cells in the somatosensory cortex, there was no interaction between the genotype and the treatment, and pairs of groups were analyzed in an unpaired, two-tailed Student’s t-test. * p<0.05


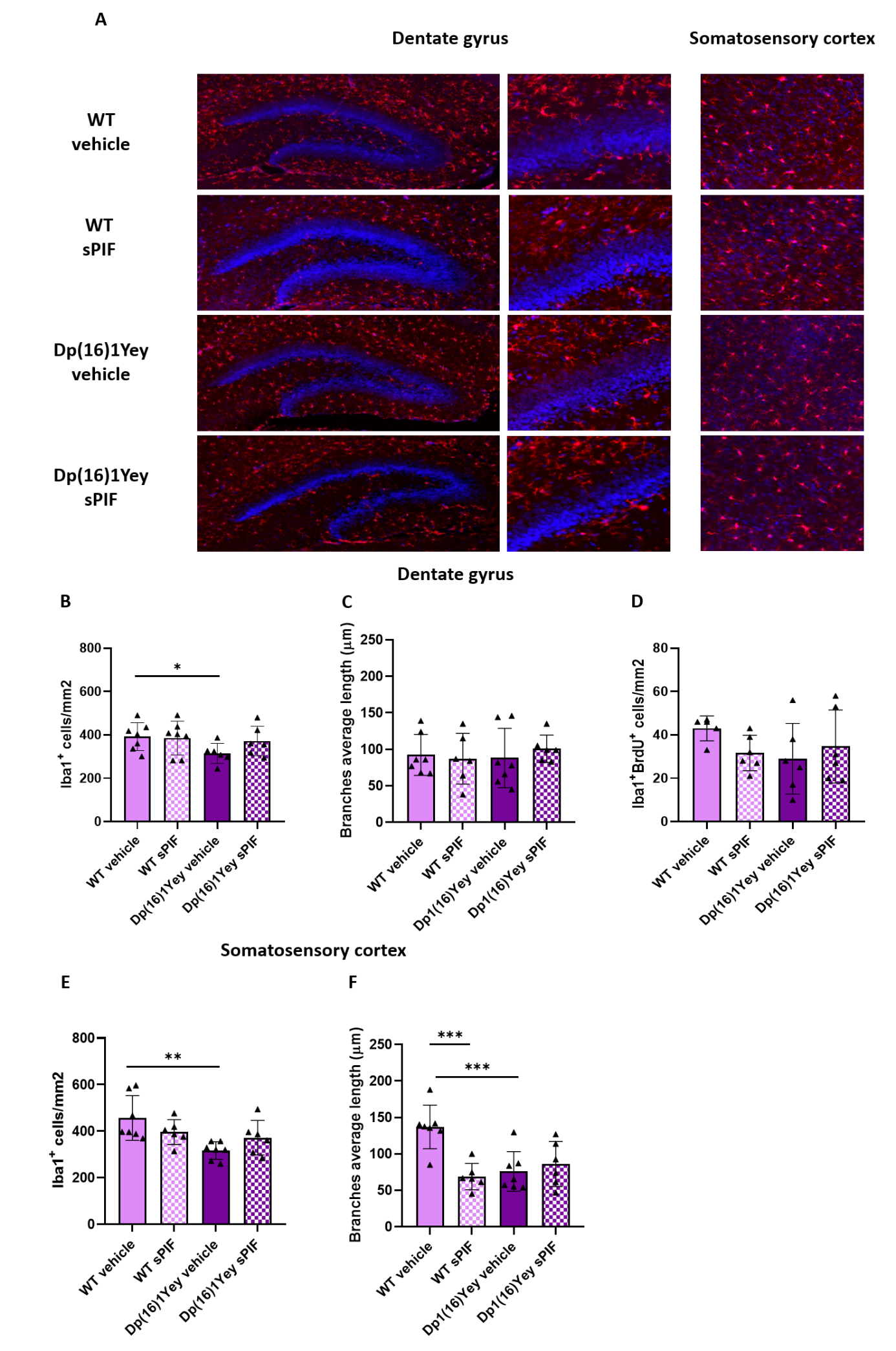


**Figure S1**


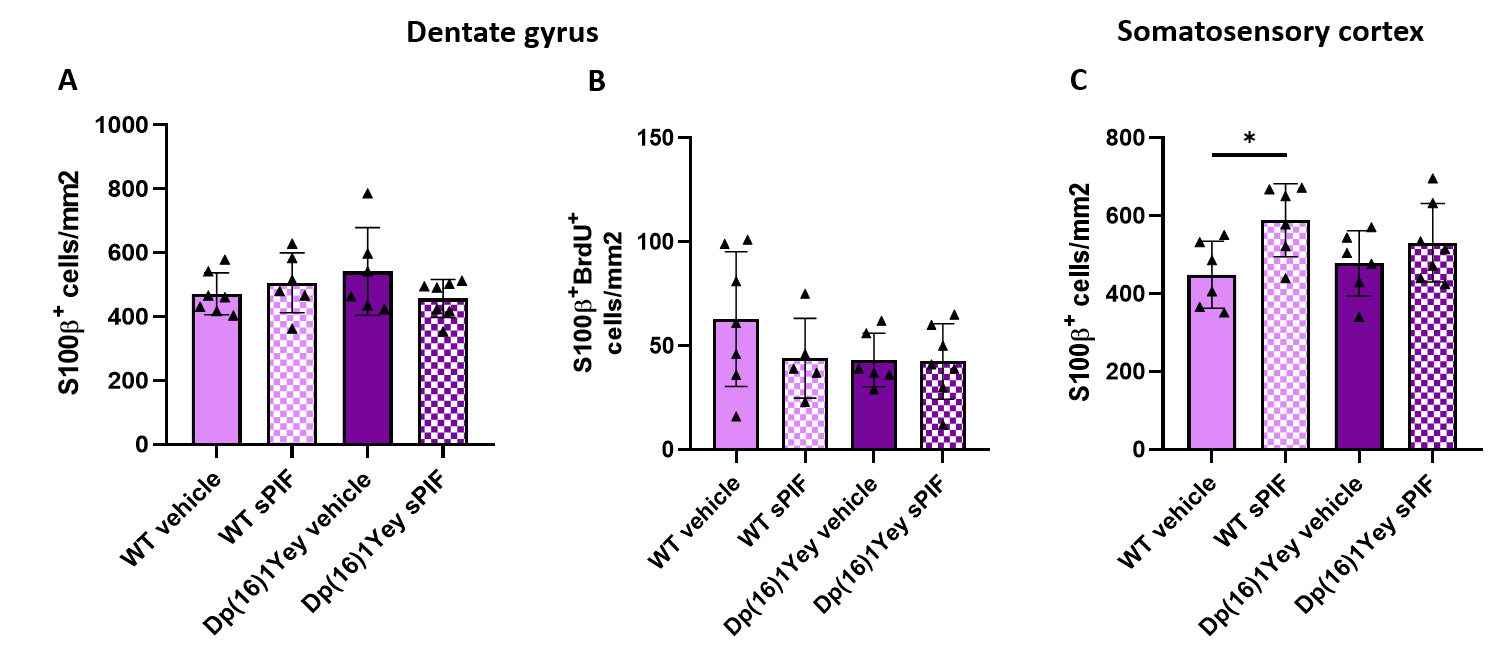


**Figure S2**
